# Ferroelectricity in underdoped La-based cuprates

**DOI:** 10.1038/srep15268

**Published:** 2015-10-21

**Authors:** Z. Viskadourakis, S. S. Sunku, S. Mukherjee, B. M. Andersen, T. Ito, T. Sasagawa, C. Panagopoulos

**Affiliations:** 1 Crete Center for Quantum Complexity and Nanotechnology, University of Crete, Heraklion 71003, Greece; 2 IESL-FORTH, Vassilika Vouton, Heraklion 71110, Greece; 3 Division of Physics and Applied Physics, School of Physical and Mathematical Sciences, Nanyang Technological University, 637371 Singapore; 4 Niels Bohr Institute, University of Copenhagen, Copenhagen DK-2100, Denmark; 5 National Institute of Advanced Industrial Science and Technology, Tsukuba, Ibaraki 305-8562, Japan; 6 Materials and Structures Laboratory, Tokyo Institute of Technology, Kanagawa 226-8503, Japan; 7 Department of Physics, University of Crete, Heraklion 71003, Greece

## Abstract

Doping a “parent” antiferromagnetic Mott insulator in cuprates leads to short-range electronic correlations and eventually to high-T_c_ superconductivity. However, the nature of charge correlations in the lightly doped cuprates remains unclear. Understanding the intermediate electronic phase in the phase diagram (between the parent insulator and the high-T_c_ superconductor) is expected to elucidate the complexity both inside and outside the superconducting dome, and in particular in the underdoped region. One such phase is ferroelectricity whose origin and relation to the properties of high-T_c_ superconductors is subject of current research. Here we demonstrate that ferroelectricity and the associated magnetoelectric coupling are in fact common in La-214 cuprates namely, La_2-x_Sr_x_CuO_4_, La_2_Li_x_Cu_1-x_O_4_ and La_2_CuO_4+x_. It is proposed that ferroelectricity may result from local CuO_6_ octahedral distortions, associated with the dopant atoms and clustering of the doped charge carriers, which break spatial inversion symmetry at the local scale whereas magnetoelectric coupling can be tuned through Dzyaloshinskii-Moriya interaction.

The phase diagram of the high temperature superconducting cuprates has been under extensive investigation since their discovery, nearly three decades ago^1^. In addition to superconductivity, a variety of ground states have been proposed and partly realized upon charge carrier doping. For example, the parent compound La_2_CuO_4_, which is an antiferromagnetic (AF) Mott insulator with Néel temperature T_N _= 325 K^2^, is known to exhibit a short-range glassy phase^3^,^4^,^5^,^6^,^7^ and subsequently diagonal stripe order upon doping with e.g. Sr (La_2-x_Sr_x_CuO_4_)^8^,^9^,^10^. Recently, research has refocused on the highly underdoped cuprates^11^,^12^. However, the evolution of the electronic ground state with the very first added charge carriers, in particular in the search for a possible broken symmetry associated with an exotic ground state and the relevant consequences on the anomalous normal state properties remains unresolved.

Although earlier efforts had in fact suggested that ferroelectricity could be present in La_2_CuO_4+x_ and YBa_2_Cu_3_O_6+x_,^13^,^14^ a low temperature ferroelectric (FE) phase with an associated magnetoelectric (ME) coupling was observed only recently in lightly oxygen doped La_2_CuO_4+x_ (T_N _= 320 K)^15^. Ferroelectricity is characterized by broken spatial inversion symmetry and typically emerges in the absence of mobile charge carriers. In the case of lightly oxygen doped La_2_CuO_4+x_^15^ it was proposed that the non-stoichiometric oxygen ions occupy interstitial positions in the La_2_CuO_4_ unit cell, causing a displacement in the apical oxygen ions of the CuO_6_ octahedra, which are the building blocks of the La_2_CuO_4_ unit cell. Therefore, local-scale structural CuO_6_ distortions take place breaking spatial inversion symmetry and resulting in the formation of local electric dipoles^16^,^17^. These dipoles localize around the oxygen interstitials forming charge clusters, which couple and freeze at low temperature giving rise to a measurable polar state.

Other microscopic mechanisms have also been proposed. These include a model based on polaron formation around the dopant atoms^18^, a purely magnetic origin associated with Dzyaloshinskii-Moriya (DM) interaction leading to local structural distortions and concomitant local broken inversion symmetry^19^, a proposal for the emergence of electric polarization due to the formation of magnetic vortex/anti-vortex pairs at the edges of oriented stripe segments^20^, and a bound cluster model^21^,^22^. The presence of the ME effect in La_2_CuO_4+x_ has also been studied by Landau theory, which includes a bi-quadratic coupling between the electric polarization and the magnetic order^23^,^24^. At present, there is no consensus on the origin of the FE phase in these materials. A central question is whether ferroelectricity is universal in these doped Mott insulators and for example, if it is present in La_2_CuO_4_ when different dopant ions are introduced into the lattice. To shed light on the nature of ferroelectricity and magnetoelectricity in the La_2_CuO_4_ system, it is important to study other members of the La-214 cuprate family, especially the case where charge carriers originate from Sr and Li doping. These ions occupy stoichiometric positions in the La-Cu-O unit cell and therefore, no dipole moments are directly associated with the dopant sites, in contrast to the case of interstitial excess oxygen ions in La_2_CuO_4+x_.

Here, we report measurements of the electric polarization on Sr and Li doped La_2_CuO_4_ single crystals. We show that La_1.999_Sr_0.001_CuO_4+y_ exhibits distinct FE behavior along different crystallographic directions. A similar behavior is observed in lightly oxygen doped La_2_CuO_4+x_ (these are different samples to those investigated in ref. 15) for direct comparison to La_1.999_Sr_0.001_CuO_4+y_. We find that the magnetic field dependence of the electric polarization is anisotropic and tunable through the DM interaction. In La_2_Li_x_Cu_1-x_O_4_ (x = 0.01 and x = 0.04), the measured electric polarization is in the μC cm^−2^ range i.e., several times higher than for La_1.999_Sr_0.001_CuO_4+y_ and La_2_CuO_4+x_. Furthermore, the electric polarization in La_2_Li_x_Cu_1-x_O_4_ is tunable by an applied magnetic field in a manner similar to La_1.999_Sr_0.001_CuO_4+y_ and La_2_CuO_4+x_. These results taken together demonstrate that the FE phase is present in all the underdoped La-214 cuprates we have investigated so far. We propose ferroelectricity may originate from a mechanism that breaks inversion symmetry by local structural distortions of the CuO_6_ octahedra, that is induced by the presence of the dopant ions and clustering of the added holes.

## Results and Discussion

**Figure 1:**
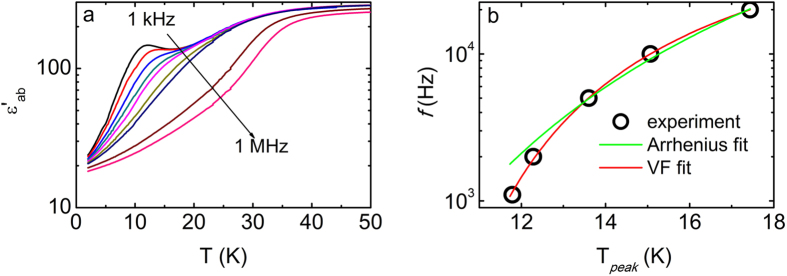
In-plane dielectric permittivity for La_1.999_Sr_0.001_CuO_4+y_. **(a)** ε′_ab_ as a function of temperature (for various frequencies). (**b**) *f* vs. T_*peak*_ graph as extracted from panel (a) (black solid circles). Green and red solid lines depict the Arrhenius and Vogel-Fulcher (VF) fits, respectively.

Figure 1a shows the in-plane dielectric permittivity ε′_ab_(T) for La_1.999_Sr_0.001_CuO_4+y_. At high temperatures, a step-like decrement is observed at all measured frequencies. This decrement shifts to higher temperature with increasing frequency *f*, indicative of a common dielectric relaxation process. At low frequencies, an additional dielectric peak develops which shifts to higher temperatures and is suppressed with increasing *f*. Such a peak in ε′_ab_ may be either due to an intrinsic charge relaxor characterized by a diffused phase transition, or due to spurious effects arising from the electrical contacts, such as extrinsic Maxwell-Wagner effects^25^,^26^.

To clarify the intrinsic character of the dielectric peaks, ε′_ab_(T) measurements were repeated several times with renewed contacts giving consistent results in the temperature regime around the peaks. Furthermore, ε′_ab_(T) measurements were performed by varying the contact area as well as upon combined dc and ac electric fields^25^, showing no difference in the measured curves. Additionally, both the temperature where the dielectric peak occurs - T_*peak*_ - and the magnitude of the permittivity at the peak position ε′_*peak*_ support the intrinsic origin of ferroelectricity since they cannot be described by the empirical relations reported for a pseudo-FE relaxor^15^,^25^,^26^.

Figure 1b shows the *f* dependence of T_*peak*_ as extracted directly from Fig. 1a. Our attempt to fit the experimental data using the Arrhenius relation (*f = f*_*o*_*exp [–E*_*a*_*/k*_*B*_*T]*) was unsuccessful. On the other hand, a relaxation process due to the slowing of polar clusters can be effectively described by the Vogel-Fulcher (VF) relation *f = f*_*o*_*exp [–E*_*a*_*/k*_*B*_*(T - T*_*fr*_) ], where the characteristic freezing temperature T_*fr*_ corresponds to the temperature below which the polar clusters freeze^27^. Applying the VF relation we obtain T_*fr-ab *_= (7.3 ± 0.3) K. Similar analysis of the out-of-plane dielectric permittivity ε′_c_ gives a freezing temperature T_*fr-c *_= (8.6 ± 0.5) K (supplementary S3), suggesting an anisotropic behavior in the charge dynamics, in agreement with an earlier report^6^. Furthermore, the excellent VF fit to the experimental data adds credence to the intrinsic character of the low temperature permittivity peaks – extrinsic effects would have been described by an Arrhenius law^27^,^28^. Thus, the above mentioned observation is likely due to a FE relaxor characterized by a diffused phase transition and the freezing of a short-range cluster-like order. In fact electric polarization may emerge below T_*fr*_, in relaxor ferroelectrics^29^.

**Figure 2:**
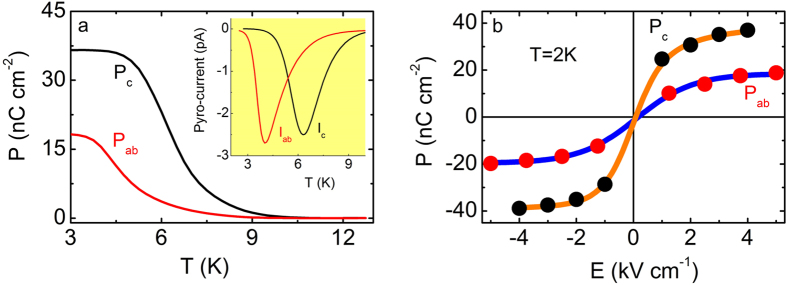
Electric polarization for La_1.999_Sr_0.001_CuO_4+y_. (**a**) In-plane P_ab_ (red line) and out-of-plane P_c_ (black line) electric polarization as a function of temperature. **Inset.** The corresponding pyroelectric current curves. (**b)** Polarization as a function of applied electric field (P-E) curves for P_ab_ (red solid circles) and P_c_ (black solid circles) at 2 K. Blue and orange solid lines are guides to the eye.

Figure 2a shows the in-plane, P_ab_(T), and the out-of-plane, P_c_(T), electric polarization for La_1.999_Sr_0.001_CuO_4+y_. For both orientations, the polarization increases with decreasing temperature below 10 K. Notably, P_c_ and P_ab_ exhibit distinct temperature dependences. Furthermore, corresponding as-measured pyroelectric current curves (Fig. 2a, inset) indicate the pyroelectric current local minima occur at different temperatures, namely at 4 K and 6.5 K for the in-plane and the out-of-plane orientations, respectively. A similar anisotropy has been observed in the spin-glass temperature^9^,^30^. Furthermore, the electric polarization curves reverse with reversing the polarity of the applied electric field, satisfying an essential condition for this phase to be described as ferroelectric.

In proper FE’s the onset of ferroelectricity is defined as the temperature above which there is no measurable polarization and the corresponding pyroelectric current exhibits a sharp minimum. On the other hand, in relaxor FE’s a transition occurs over a long time scale. Similarly in our data, the pyroelectric minimum is not sharp, resulting in a broad transition in the polarization with respect to temperature therefore, preventing a precise assignment of a transition temperature. We therefore assign the transition towards a FE state as the temperature where a minimum (albeit broad) occurs in the pyroelectric current. This assignment is corroborated by the fact that the temperature where the local minimum occurs is robust against both the electric field applied during cooling and the temperature sweep rate.

The electric polarization as a function of applied electric field (P - E curves) for both measured orientations is shown in Fig. 2b (T = 2 K). P changes with E, resulting in S-like P-E curves for both measured orientations. However, the out-of plane component exhibits higher P values than the in-plane counterpart (namely P_ab _= 18 nC cm^−2^ and P_c _= 36 nC cm^−2^ at 2 K). In contrast, the in-plane component changes relatively smoothly with E. The above observation suggests a sizable anisotropy between in-plane and out-of-plane electric polarization, possibly linked to the anisotropy in the charge dynamics discussed earlier.

To confirm the different trends and elucidate the effect of Sr doping in La_1.999_Sr_0.001_CuO_4+y_ (T_N _= 312 K) we performed electric polarization measurements on lightly oxygen-doped La_2_CuO_4+x_ with T_N _= 313 K. Compared to our previous studies^15^, the La_2_CuO_4+x_ samples investigated here exhibit a lower T_N_, indicative of a higher excess oxygen concentration and therefore, higher charge carrier concentration. Approximate estimates of the excess oxygen and carrier concentration for the La_2_CuO_4+x_ samples studied here are 0.40 ± 0.08 mol% and ~10 ^18^cm^−3^, respectively^31^, which are significantly lower than corresponding values in ref. 15. Similarly, for La_1.999_Sr_0.001_CuO_4+y_ we obtain n ~ 10^18^ cm^−3^ and 0.43 ± 0.08 mol%. Notably, the Sr doping level is sufficiently low to affect the Néel transition, thus the suppression of T_N_ is likely due to excess oxygen^32^,^33^.

**Figure 3:**
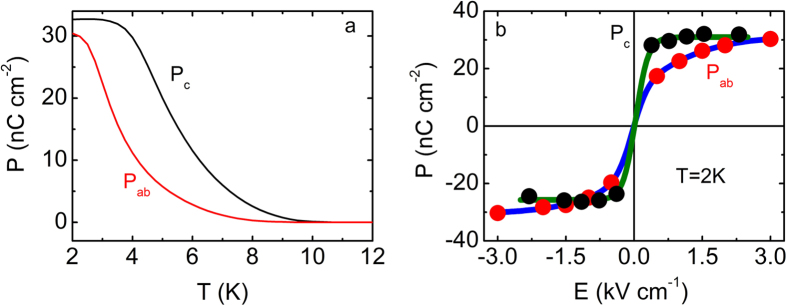
Electric polarization for La_2_CuO_4+x_ (T_N _= 313 K). (**a**) In-plane P_ab_ (red line) and out-of-plane P_c_ (black line) electric polarization as a function of temperature. **(b)** Polarization as a function of applied electric field (P-E) for P_ab_ (red symbols) and P_c_ (black symbols) at 2 K. Dark green and blue lines are guides to the eye.

Figure 3a shows the temperature dependence of P_ab_ and P_c_ for La_2_CuO_4+x_ (T_N_ = 313 K). Both P_ab_ and P_c_ increase with decreasing temperature however, P_c_ exhibits a distinct temperature dependence compared to P_ab_(T). Namely, a change in slope is observed at 5 K and 3 K for the out-of-plane and the in-plane orientation, respectively. (These temperatures differ to those for the La_2_CuO_4+x_ samples reported in ref. 15 where T_FE _= 4.5 K for both the in-plane and-out of plane directions. Notably, the anisotropy is reduced with increasing the Neel temperature, as shown for the electric conductivity and the dielectric constant^34^,^35^). At T = 2 K, P_c_ is almost equal to P_ab_. However, P_c_ reverses almost immediately with reversing the electric field (E) and saturates at low fields (Fig. 3b). On the other hand, P_ab_ increases gradually with E showing an S-like behavior and tends to saturate at high values of E. This trend is qualitatively similar to the behavior observed in La_1.999_Sr_0.001_CuO_4+y_, indicating that anisotropy is an intrinsic property of the system. Comparison between La_2_CuO_4+x_ and La_1.999_Sr_0.001_CuO_4+y_, reveals distinct differences in the dielectric permittivity, FE transition temperatures and anisotropy (supplementary table ST1). We also measured La_1.998_Sr_0.002_CuO_4+y_ however, the material was not sufficiently insulating to permit transport measurements of the electric polarization. Hence, although possible excess oxygen may still affect our observations in La_1.999_Sr_0.001_CuO_4+y_, the above results indicate Sr doping influences the FE behavior of La_2_CuO_4+x_ and is distinct from oxygen doping.

We now turn to the effect of the applied magnetic field. The spins in the AF phase of La-214 are weakly canted out-of-plane due to the presence of a finite DM interaction^36^. Spin canting causes a weak ferromagnetic (WF) moment along the c-axis in each CuO_2_ plane, although the opposite spin canting in alternate planes leads to a net cancellation of the WF moments. By applying an external magnetic field along the c-axis, the WF moments can be aligned in the same direction above a critical magnetic field H_cr_, which for La_1.999_Sr_0.001_CuO_4+y_ is 6 T (supplementary S5).

**Figure 4:**
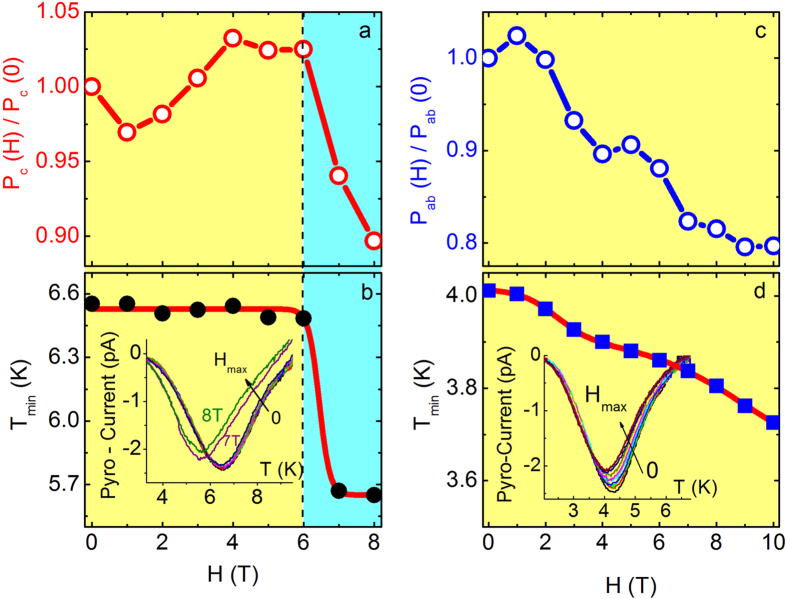
Magnetoelectric coupling in La_1.999_Sr_0.001_CuO_4+y_. (**a**) P_c_(H)/P_c_(0) vs. H at T = 2 K (H||c and E||c). The black dashed line depicts the critical magnetic field above which the sample enters the WF state (light blue region). (**b)** T_min_ vs. H curve. Solid black symbols correspond the temperatures where the pyrocurrent minima occur for each magnetic field (inset graph). Red solid line is a guide to the eye. (**c)** P_ab_ (H)/P_ab_ (0) vs. H, at T = 2 K (H||ab and E||ab). (**d)** T_min_ vs. H graph. Solid blue symbols correspond the temperatures where the pyrocurrent minima occur for each magnetic field (inset graph). Red solid line is a guide to the eye.

Figure 4a shows P_c_ (E||c) as a function of applied magnetic field (H||c). P_c_ decreases abruptly above H_cr _= 6 T. Furthermore, the temperature where the pyroelectric current minimum occurs - T_min_ (Fig. 4b) - exhibits a strong decrease above H_cr_, indicating a suppression in the onset of FE as the material enters the WF state (similar changes are also observed for H||c and E^c). On the other hand, P_ab_ (E||ab) decreases progressively with increasing H||ab (Fig. 4c), while T_min_ shifts smoothly to lower temperatures with increasing H (Fig. 4d - similar changes are observed for E^ab and H||ab). Hence, ferroelectricity in La_1.999_Sr_0.001_CuO_4+y_ is coupled to the underlying AF structure and is influenced by the DM interaction in a manner similar to recent observations for La_2_CuO_4+x_^15^,^23^,^24^.

The similarity in the measured FE state among La_2_CuO_4+x_ and La_1.999_Sr_0.001_CuO_4+y_ cuprates is notable even though oxygen and Sr doping do not affect the CuO_6_ structure in the same way - the excess oxygen take non-stoichiometric positions, while Sr ions substitute for La - suggesting a common origin of ferroelectricity in these materials. Because Sr and oxygen dopants take positions outside the oxygen octahedra, it is important to study the evolution of ferroelectricity in the La-214 family of cuprates using also Li doping, which directly replaces Cu ions in the CuO_6_ octahedra. Among other dopants, such as Mg and Zn which can be used to replace Cu, Li ions exhibit the largest ionic radius (0.76 Å) compared to 0.74 Å for Zn, 0.72 Å for Mg and 0.73 Å for Cu. Furthermore, La_2_Li_x_Cu_1-x_O_4_ remains insulating for Li doping up to 4%^37^,^38^, allowing pyrocurrent measurements even at relatively high carrier concentration.

**Figure 5:**
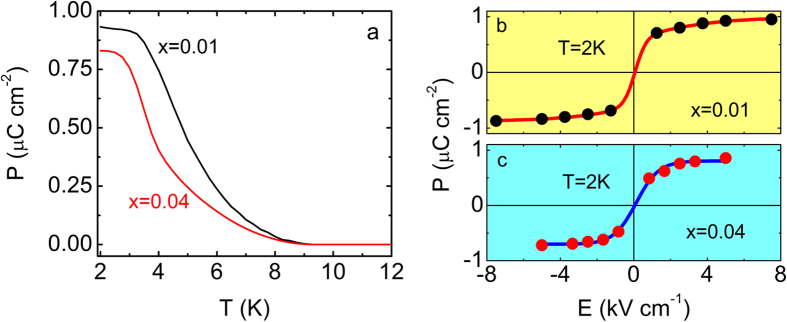
Electric polarization for La_2_Li_x_Cu_1-x_O_4_. **(a)** P_c_ as a function of temperature for La_2_Li_x_Cu_1-x_O_4_ with x = 0.01 (black solid line) and x = 0.04 (blue solid line). P-E curves for **(b)** x = 0.01 and **(c)** x = 0.04 at 2 K are also shown. Red and blue solid lines are guides to the eye.

Figure 5a shows the temperature dependence of P_c_ for La_2_Li_x_Cu_1-x_O_4_ (x = 0.01 and x = 0.04). In both cases P_c_ increases with decreasing temperature, below ~9 K. A change in slope in the electric polarization occurs at 5 K for x = 0.01 and at 3.5 K for x = 0.04. These temperatures are also comparable to the values of charge cluster freezing temperatures reported by Park *et al.*^39^ suggesting a link between charge glassines and the onset of ferroelectricity in La-214 cuprates. The corresponding P-E curves (Fig. 5b-5c) reveal a behavior similar to that observed in La_1.999_Sr_0.001_CuO_4+y_ and La_2_CuO_4+x_. Furthermore, the polarization is remarkably large namely, 900 nC cm^−2^ for x = 0.01 and 800 nC cm^−2^ for x = 0.04. However, for the in-plane orientation, ac conductivity analysis reveals a dominant in-plane electrical conductivity in the low temperature regime preventing the measurement of pyroelectricity^6^.

**Figure 6:**
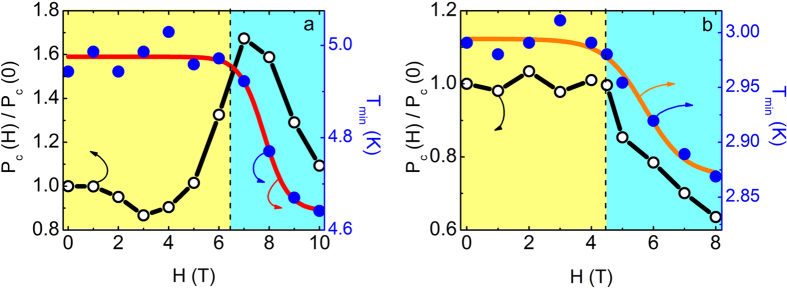
Magnetoelectric coupling in La_2_Li_x_Cu_1-x_O_4_. Effect of the applied magnetic field to the electric polarization (**a)** for La_2_Li_0.01_Cu_0.99_O_4_ and (**b)** for La_2_Li_0.04_Cu_0.96_O_4_, respectively. In both cases T = 2 K, H||c and E||c. T_min_ vs. H is also demonstrated in correspondence to the electric polarization. The black dashed line indicates the critical field above which the samples enter the WF state (light blue region). Red and orange solid lines are guides to the eye.

Figure 6 shows the magnetic field dependence of P_c_ for La_2_Li_x_Cu_1-x_O_4_ (x = 0.01 and x = 0.04) at T = 2 K (E||c and H||c). For x = 0.01, P_c_ is enhanced below H_cr _= 6.5 T, increasing almost 1.7 times its zero-field value (Fig. 6a) before being suppressed. It has been suggested this may be due to a DM induced magnetoelectric coupling^23^,^24^. For x = 0.04 (H_cr _= 4 T) P_c_ is roughly constant up to H_cr_, while it decreases when a WF state sets in (Fig. 6b). Moreover, T_min_ decreases above H_cr_ for both samples, similar to La_1.999_Sr_0.001_CuO_4+y_. Therefore, La_2_Li_x_Cu_1-x_O_4_ behaves qualitatively similar to La_1.999_Sr_0.001_CuO_4+y_ and La_2_CuO_4+x_ indicating ferroelectricity is a common ground state in the La-214 cuprates studied so far, with an associated magnetoelectricity tunable by DM interaction.

According to the scenario proposed earlier, in La_2_CuO_4+x_ charge clusters may give rise to ferroelectricity^15^,^23^,^24^. However, as mentioned above, Sr and Li dopant ions occupy stoichiometric positions in the La-Cu-O unit cell and therefore, no dipole moments are directly associated with the dopant sites, in contrast to the case of interstitial excess oxygen ions in La_2_CuO_4+x_. Although we cannot rule out alternative mechanisms for the emergence of ferroelectricity, here we propose that ferroelectricity could emerge from charge clustering together with CuO_6_ distortions^21^,^22^ induced by dopant ions. In particular, it has been shown^22^ that charges of dopant ions and apex oxygen atoms are combined with many-body screening effects and bind clusters of four holes. The asymmetrically distributed charge clusters break inversion symmetry locally and form randomly distributed dipoles at high temperatures. Below a temperature that depends upon the size and density of the dipolar clusters, these randomly oriented dipoles begin to align in different regions within the sample into a long range FE state. Note that the cluster size usually depends on doping^21^, and the clusters evolve anisotropically first along the ab-plane and then slowly along the c-axis. The observation of a P - E response in both measured directions for Sr and oxygen doped samples, adds credence to the presence of dipolar clusters with different directional orders. This scenario has similarities with the model proposed by Daoud-Aladine *et al.*^40^ and by D. V. Efremov *et al.*^41^, which could also be applied to non-metallic cuprates. In particular, a local charge ordering pattern such as formation of Zener polarons could lead to a locally broken inversion symmetry state. The phase diagram of high-T_c_ cuprates is known to contain both site- and bond- centered charge ordered states^41^ and they could potentially survive within short range charge clusters at lower doping levels.

In support of the above proposed scenario^21^,^22^, theoretical and experimental evidence for both Sr and oxygen-doped La_2_CuO_4_ indicate the presence of local octahedral distortions correlated to charge inhomogeneities^42^,^43^,^44^,^45^,^46^,^47^. Cordero *et al.* reported the development of a significant tilt mode associated with oxygen octahedral distortion below T = 10 K from anelastic spectroscopy measurements on nearly undoped La_2-x_Sr_x_CuO_4_ crystals^17^. It was argued that these tilt modes are due to fluctuating low temperature tetragonal structures at domain walls of the low temperature orthorhombic lattice and are coupled to hole clusters. Additionally, a non-centrosymmetric monoclinic distortion in the CuO_6_ octahedra in orthorhombic La_2-x_Sr_x_CuO_4_ and La_2_CuO_4+x_ has also been observed^16^. Notably, ferroelectricity sets in when transitions from centrosymmetric to non-centrosymmetric phases occur e.g. BaTiO_3_ undergoes a cubic (centrosymmetric) to tetragonal (non-centrosymmetric) phase transition at 120 ^o^C resulting to the onset of ferroelectricity below this temperature. The presence of non-centrosymmetric octahedral distortions in orthorhombic La_2-x_Sr_x_CuO_4_ and La_2_CuO_4+x_ may therefore explain the quantitatively similar polar behavior observed in these materials.

In the case of Li-doped La_2_CuO_4_ the polar behavior may also be associated to the local-scale distortions combined with charge clustering. Li atoms are located at the center of the CuO_6_ octahedra resulting to an increase in the distance between Li ions and the apical oxygen atoms^38^, and a corresponding tetragonal distortion^42^ (notably, tetragonal structures can be non-centrosymmetric or polar in some cases^47^). Moreover, the apical oxygen movement induces a correlated displacement of the bonded La atoms, giving rise to distortions which could locally destroy the spatial symmetry. We note however, further investigation is necessary to narrow down the possibilities for the physical mechanism driving our observations^15^,^18^,^19^,^20^,^21^,^22^,^23^,^24^.

In summary, we report evidence for the presence of FE and ME coupling in underdoped La-214 cuprates, namely La_2_CuO_4+x_, La_1.999_Sr_0.001_CuO_4+y_ and La_2_Li_x_Cu_1-x_O_4_ (x = 0.01 and x = 0.04). For both Sr and oxygen doped samples we observed distinct electric polarization behavior along the in-plane and out-of-plane crystallographic orientations. In all cases the electric polarization is strongly influenced by DM interaction. For Li-doped samples electric polarization values of the order of μC cm^−2^ are obtained. Considering the above experimental evidence, the FE order and its related magnetoelectricity appear to be a generic property of La-214 cuprates.

## Methods

### Crystal growth

The experiments were performed on twinned single crystals. La_1.999_Sr_0.001_CuO_4+y_ crystals were grown using the laser-diode heated floating zone method^48^. High purity La_2_O_3_ (99.99%), SrCO_3_ (99.9%) and CuO (99.9%) were used as starting materials. Sr ions take La stoichiometric positions and thus the Sr concentration (x = 0.001) is higher than the purity of La_2_CO_3_. On the other hand, the Sr concentration is comparable to the purity of CuO. Furthermore, the crystals were carefully annealed at 1000 ^o^C for 20 h under 1 ppm O_2_ - Ar flowing atmosphere. The crystal axes were determined using the x-ray Laue backscattering technique. The sharp antiferromagnetic transition at 312 K (see supplementary section S1) is suggestive of homogeneous distribution of the dopant ions (either Sr or excess oxygen ions) in La_1.999_Sr_0.001_CuO_4_ since an inhomogeneous distribution would cause either the suppression, broadening of the AF peak at 312 K, or the presence of secondary magnetic peaks below 312 K. To overcome the difficulty in quantifying experimentally the very low Sr concentration, we synthesized (crystal growth and annealing) oxygen doped La_2_CuO_4+x_ using the same method as for La_1.999_Sr_0.001_CuO_4+y_. The agreement in the magnetic transition (supplementary S1) indicates a similar excess oxygen doping in both materials; very small Sr doping is not expected to influence the magnetic transition temperature of La_1.999_Sr_0.001_CuO_4+y_^32^,^33^. Therefore, a possible difference in the transport properties between La_2_CuO_4+x_ and La_1.999_Sr_0.001_CuO_4+y_ would be due to Sr doping. La_2_Cu_1-x_Li_x_O_4_ single crystals were grown using the conventional Lamp Heated Floating Zone Technique^37^. High purity La_2_O_3_ (99.999%), Li_2_CO_3_ (99.99%) and CuO (99.99%) were used as starting materials to achieve the lowest possible impurity level. The crystal axes were again determined by the x-ray Laue backscattering technique. The samples were annealed at 900 ^o^C for 48 h in flowing Ar atmosphere. The Li concentration was estimated from x-ray diffraction measurements of the lattice constants^38^. All crystals were cut appropriately and pairs of plate-like samples were extracted. Each pair consists of a sample with the thinnest direction along the c-axis and another with the thinnest direction along the ab-plane.

### Magnetic measurements

The magnetization of the samples was measured using a commercial MPMS Quantum Design SQUID magnetometer, in Heraklion. The La_1.999_Sr_0.001_CuO_4+y_ crystals exhibit T_N _= 312 K, whereas for LaCuO_4+x_ T_N _= 313 K. For La_2_Li_x_Cu_1-x_O_4_ crystals, T_N _= 255 K (x =  0.01) and T_N _= 150 K (x = 0.04).

### Impedance and pyroelectric measurements

The impedance and loss of the samples were measured using an LCR meter in the frequency range 21 Hz – 2 MHz. The dielectric permittivity was extracted as described elsewhere^6^. Electric polarization measurements were performed using two different home-built experimental stations in Heraklion and Singapore employing the pyrocurrent technique. To measure accurately the electric polarization of our samples we employ the following protocol. The measurement process begins with the sample well above its FE transition temperature (paraelectric state). An electric field is applied and the sample is cooled to 2 K. The electric field is then removed to fulfill the conditions described earlier. The sample is then heated at 3 K/min. During this process, time, temperature and current are measured at a constant interval of 0.5 seconds. The sample is heated to well above its FE transition temperature. In the case of measuring the pyrocurrent in the presence of an applied magnetic field, we apply the magnetic field as soon as the sample reaches 2 K and before removing the applied electric field (Zero Magnetic Field Cooling). Finally, the P vs. E curves are obtained by measuring the pyroelectric current as a function of temperature, for various applied electric voltages. Further information regarding the pyrocurrent data processing can be found in the supplementary (section S2).

## Additional Information

**How to cite this article**: Viskadourakis, Z. *et al.* Ferroelectricity in underdoped La-based cuprates. *Sci. Rep.* **5**, 15268; doi: 10.1038/srep15268 (2015).

## Supplementary Material

Supplementary Information
