# Supplementary material for: Ferroelectricity in underdoped La-based cuprates
Source: Sci Rep. 2015 Oct 21;5:15268. doi: 10.1038/srep15268 (PMC4614081; doi:10.1038/srep15268)
Supplement: Supplementary Information [file srep15268-s1.pdf]

Ferroelectricity in underdoped La-based cuprates

Z. Viskadourakis, S. S. Sunku, S. Mukherjee, B. M. Andersen, T. Ito, T. Sasagawa and C. Panagopoulos

Supplementary Information

S1. Magnetization measurements

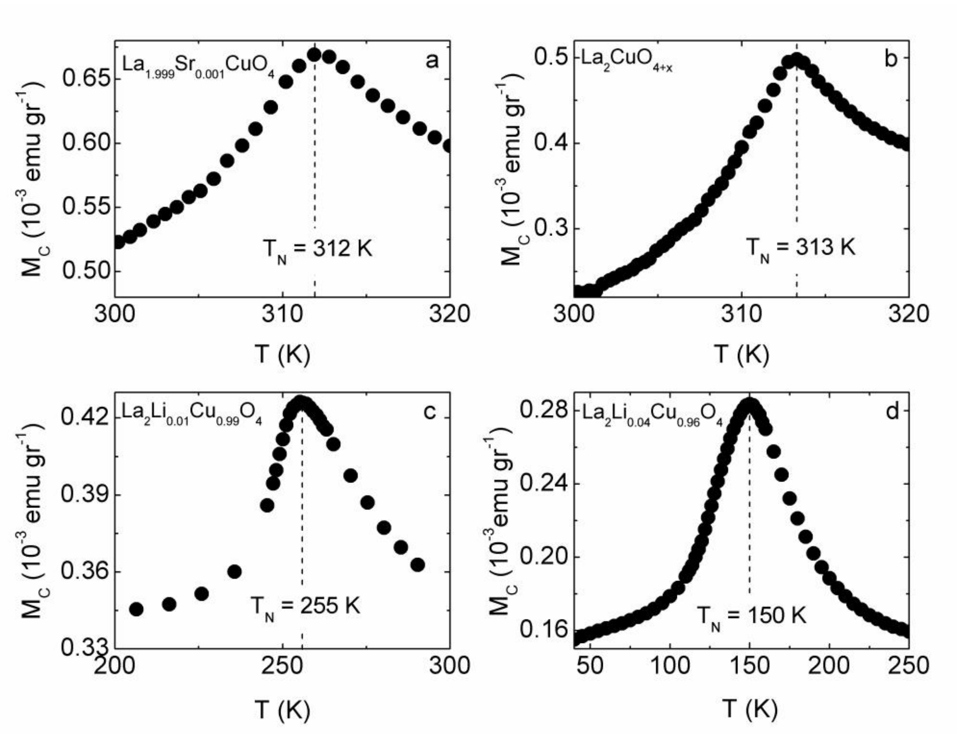

Figure S1: *Bulk magnetization with the magnetic field applied along the c-axis (H = 1 kG) for* ***a.*** *$La_{1.999}Sr_{0.001}CuO_{4+y}$,* ***b.*** *lightly oxygen-doped La_2CuO_{4+x},* ***c.*** *$La_2Li_{0.01}Cu_{0.99}O_4$ and* ***d.*** *$La_2Li_{0.04}Cu_{0.96}O_4$. The observed peaks correspond to the Néel transition temperatures (T_N). The corresponding magnetization is comparable to earlier studies.[1, 2]*

S2. Pyrocurrent data processing

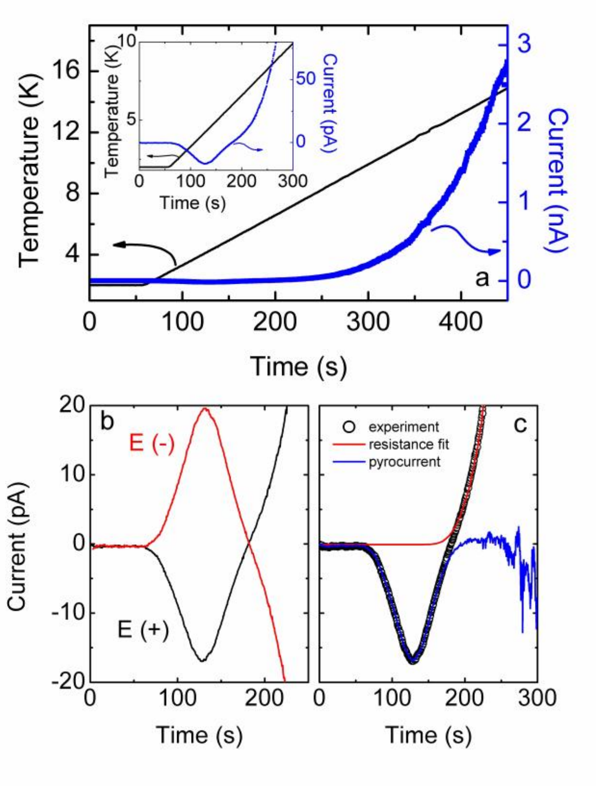

***Figure S2**: **a.** Electric current (blue solid line) and temperature (black solid line) measured during the experimental process. The inset of the graph depicts a detailed picture of the measured current. A local current minimum is recorded. **b.** The local minimum changes sign with respect to the electric field reversal. **c**. The measured electric current (black open circles) can be processed to extract the pyroelectric signal (blue solid line) to determine the electric polarization of the sample. The red solid line represents the resistive part of the measured current.*

A typical measured current-vs.-time data set is shown in fig. S2a. For T > 7 K the current increases steeply with increasing temperature indicating the conductive character of the sample. Although V = 0 during the measurement process, a small but unavoidable voltage arising from the voltage source is still applied to the circuit causing the observed resistive behavior - the level of this voltage depends on the current measurement range used in the current meter and can be between μV and 200 mV. However, in the temperature range 2 K – 6 K there is a local current minimum (inset fig. S2a) and its sign is reversed (fig. S2b) upon reversing the electric field during the cooling process. Furthermore, it becomes sharper

with increasing the temperature sweep rate, suggesting its pyroelectric nature. Thus it is important to distinguish between the pyrocurrent and the resistive components of the current in order to accurately determine the electric polarization of the sample. To this point, we fit the experimental data above 8 K against a resistivity model.[3] The best fit is subtracted from the experimental data and the resulting curve is the pure pyroelectric component (fig. S2c). Beyond 250 seconds there is considerable noise due to the data processing. Thus, the pyrocurrent curve is integrated with respect to time in the range 0 - 250 seconds, and the electric polarization with respect to time is acquired. We obtain P(T) by converting time to temperature. It must be noted that temperature regimes and time scales change accordingly to the samples measured.

S3. Out-of-plane dielectric permittivity of $La_{1.999}Sr_{0.001}CuO_{4+y}$

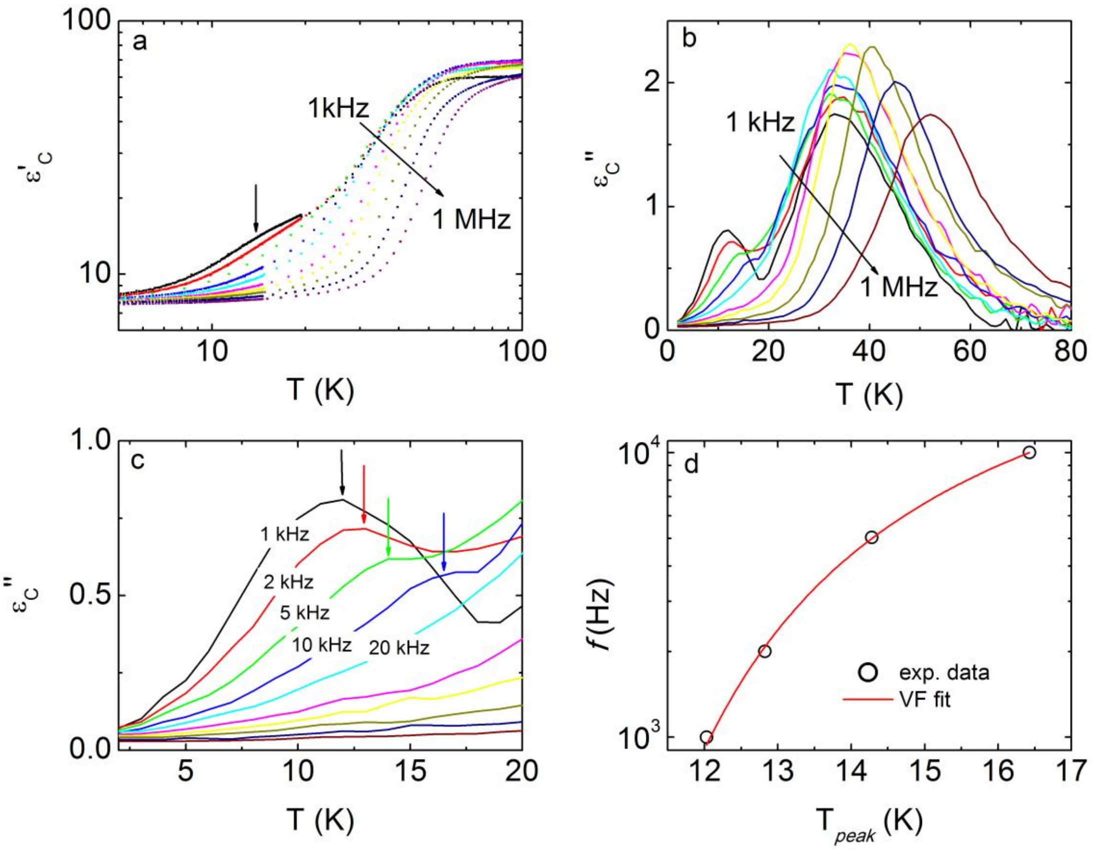

Fig. S3 a. *$\varepsilon'_c(T)$ for various frequencies for the $La_{1.999}Sr_{0.001}CuO_{4+y}$ sample. $\varepsilon'_c(T)$ exhibits a step-like behavior at ~35 K which shifts to higher temperatures with increasing f. Furthermore a light shoulder appears at ~15 K for 1 kHz, which also shifts to higher temperatures as f increases.* ***b.*** *Imaginary part of the dielectric permittivity as a function of temperature for various f. The two peaks observed correspond to the two relaxation processes obtained in the real part of the dielectric permittivity.* ***c.*** *Low temperature dielectric loss peaks (indicated by colored arrows), which correspond to the permittivity peaks observed in panel a.* ***d.*** *f vs. T_{peak} plot as extracted from panel c. The red solid line corresponds to the Vogel-Fulcher (VF) fit - T_{fr} = (8.6 ± 0.5) K.*

S4. Dielectric permittivity of the lightly oxygen-doped La_2CuO_{4+x}

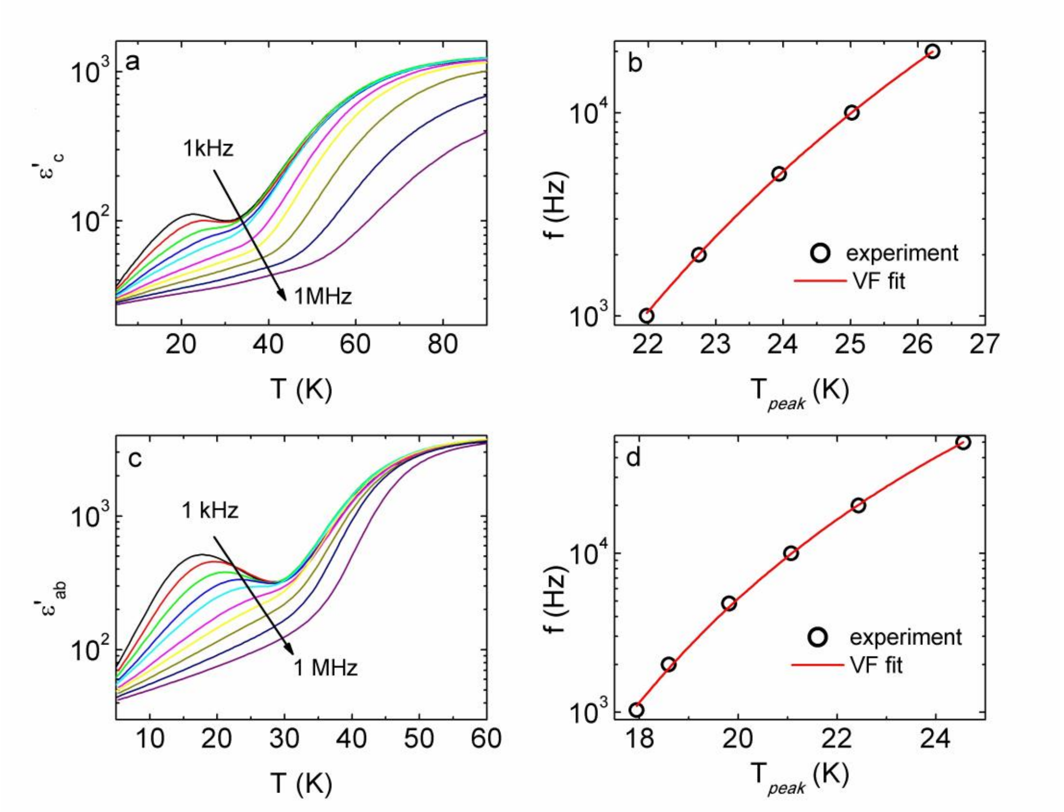

Figure S4: a. *Real part of the dielectric permittivity ε'_c as a function of temperature for lightly oxygen-doped La_2CuO_{4+x} (T_N = 313 K), measured for various frequencies. ε'_c exhibits a step-like decrement (near 60 K) with decreasing temperature and shifts to higher temperature with increasing frequency indicating a common dipolar relaxation process. Furthermore, a dielectric peak is observed at T ~ 20 K.* ***b.*** *Frequency f as a function of peak temperature T_{peak} (black open circles) extracted from panel a. The red solid line corresponds to the Vogel-Fulcher fit - $T_{fr\text{-}c}$ = (8 ± 0.5) K.* ***c.*** *Real part of the in-plane dielectric permittivity ε'_{ab} as a function of temperature for various frequencies.* ***d.*** *Vogel-Fulcher fit (red solid line) applied to the f - T_{peak} curve temperature data (black open circles) gives $T_{fr\text{-}ab}$ = (5.2 ± 0.4) K.*

S5. Magnetoresistance measurements

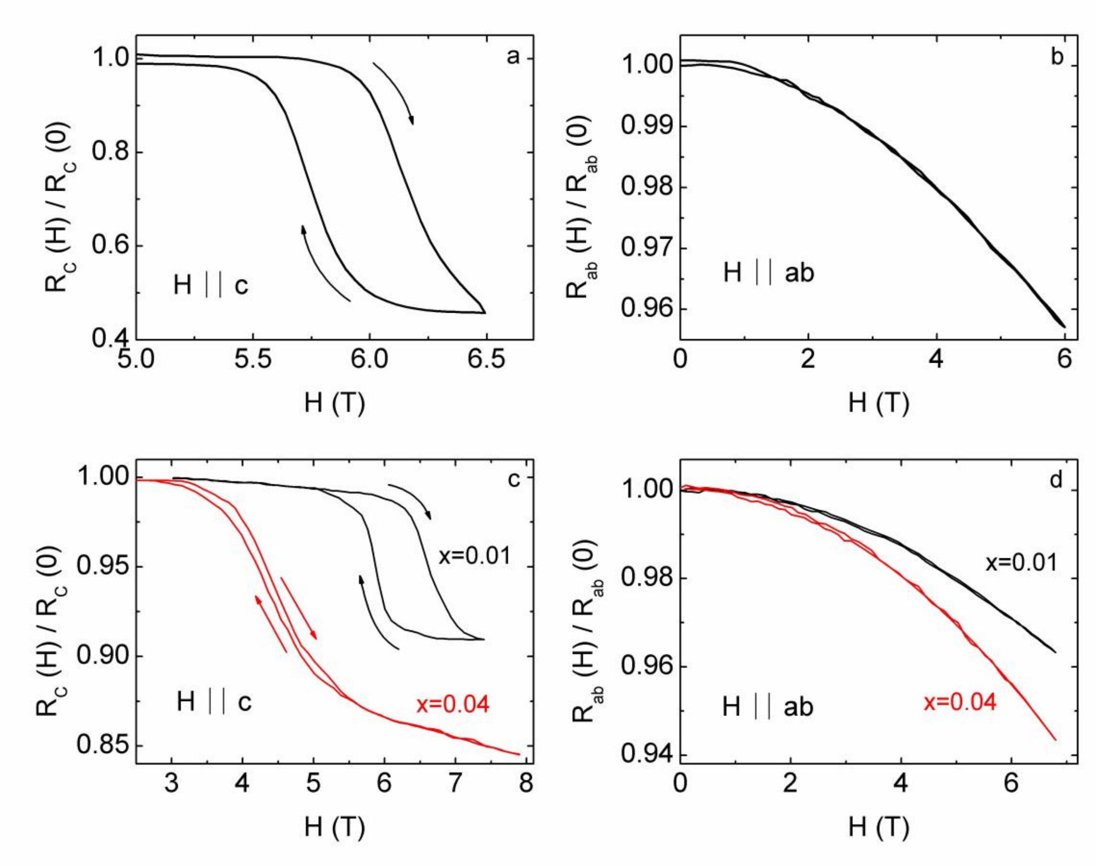

Figure S6: a. *Magnetic field dependence of the out-of-plane normalized resistivity $R_C(H)$ / $R_C(0)$ for $La_{0.999}Sr_{0.001}CuO_{4+y}$ (T = 20 K; E c and H c).* ***b.*** *In-plane normalized resistivity $R_{ab}(H)$ / $R_{ab}(0)$ as a function of applied magnetic field (T = 20 K; E ab and H ab).* ***c.*** *$R_C(H)$ / $R_C(0)$ vs. H c for $La_2Li_xCu_{1-x}O_4$ (T = 20 K; x = 0.01 and x = 0.04).* ***d.*** *$R_{ab}(H)$ / $R_{ab}(0)$ vs. H (T = 20 K; E ab and H ab) for both Li-doped samples.*

Figure S6 shows the magnetic field dependence of the normalized in-plane (fig. S6b) and out-of-plane (fig. S6a) resistivity of $La_{0.999}Sr_{0.001}CuO_{4+y}$ determined from measurements of the electric impedance at T = 20 K. For H || c (fig. S6a) we observe a first order phase transition and a corresponding hysteresis at H ~ 6 T due to the metamagnetic transition associated with Dzyaloshinskii-Moriya (DM) interactions. In La_2CuO_{4+x}, the crystal anisotropy and the DM interaction fix the easy axis for the spins to the longer of the two in-plane orthorhombic directions (the b-axis). The direction of the weak ferromagnetic (WF)

moments **L** induced by the DM interaction is fixed by the cross product **L**=**D**×$\mathbf{n_0}$ between the DM vector **D** (oriented along the shorter of the two in-plane orthorhombic directions – the a-axis) and the AF order parameter $\mathbf{n_0}$ (pointing along the b-axis) so that **L** is oriented along the c-axis, perpendicular to the CuO_2 planes of the crystal structure. A sufficiently large magnetic field applied along the c-axis can overcome the inter-plane AF coupling and induce a discontinuous spin-flop reorientation, causing the so-called WF (first order) phase transition. The critical field is reduced at high temperatures following the decrease in **L** due to thermal fluctuations in $\mathbf{n_0}$(T).[4] For H ab (fig. S5b), the magnetoresistance varies smoothly because the WF moments induce a continuous rotation of $\mathbf{n_0}$ in the bc-plane. Similar results are obtained for the Li doped samples, as shown in figs S6c and S6d.

	Estimated Carrier Concentration (cm^{-3})	Dielectric permittivity (@1kHz)	T_{FE} (K)	Pc(@2K) (nC cm^{-2})
**La_2CuO_{4+x} (T_N=320K) PRB (2012)[5]**	~ 10^{17}	~2000 (50K – out of plane)	4.5	33
		~3000 (50K – in plane)	4.5	26
**La_2CuO_{4+x} (T_N=313K) Present study**	~ 10^{18}	~1200 (100K – out of plane)	5	30
		~1300 (50K – in plane)	3.5	27
**$L_{1.999}Sr_{0.001}CuO_{4+y}$ (T_N=312K) Present study**	~ 10^{18}	~70 (100K – out of plane)	6.5	36
		~290 (50K – in plane)	4	18

Table ST1: Summarized values of the dielectric permittivity and the electric polarization for both oxygen and Sr doped La_2CuO_4 samples. Corresponding values from our previous investigation on excess oxygen doped La_2CuO_{4+x}[5] are also included.

References

[1] Sasagawa, T., Mang, P. K., Vajk, O. P., Kapitulnik, A. & Greven *Phys. Rev. B* **66** 184512 (2002).

[2] Lavrov, A. N., Ando, Y., Komiya, S. & Tsukada, I. *Phys. Rev. Lett.* **87** 017007 (2001).

[3] The Variable Range Hopping mechanism of conduction $\sigma = \sigma_o exp(T_o/T)^{-a}$ is the most suitable mechanism to explain the hopping conduction in insulating antiferromagnets.

[4] Benfatto, L and Silva Neto, M. B. *Phys. Rev. B* **74** 024415 (2006).

[5] Viskadourakis, Z. *et al. Phys. Rev. B* **85** 214502 (2012).
